# Supplementary material for: MetaRibo-Seq measures translation in microbiomes
Source: Nat Commun. 2020 Jun 29;11:3268. doi: 10.1038/s41467-020-17081-z (PMC7324362; doi:10.1038/s41467-020-17081-z)
Supplement: Supplementary file 10 — Supplementary Data 7 [file 41467_2020_17081_MOESM10_ESM.zip › File2/Confidence_High_Taxonomy/433138_out.krona.html]

Javascript must be enabled to view this page.

members
magnitude
magnitudeUnassigned
count
unassigned
taxon
rank

433138\_out

171

171
superkingdom
2

1239
phylum
1

1
class
186801

order
186802
1

family
31979
1

genus
1485
1

1
species
1262832

SRS063518\_contig\_number\_32858

170
976
phylum

200643
class
170

171549
order
170

170
family
815

31
genus
170
816

SRS011302\_contig\_number\_17310SRS013521\_contig\_number\_13793SRS013638\_contig\_number\_18875SRS014459\_contig\_number\_32477SRS015065\_contig\_number\_27423SRS015264\_contig\_number\_14524SRS015431\_contig\_number\_20596SRS015663\_contig\_number\_21264SRS016203\_contig\_number\_11371SRS016753\_contig\_number\_7955SRS019910\_contig\_number\_15640SRS020394\_contig\_number\_14360SRS045713\_contig\_number\_9489SRS050299\_contig\_number\_276SRS052697\_contig\_number\_37934SRS056259\_contig\_number\_27185SRS064276\_contig\_number\_39403SRS065176\_contig\_number\_14301SRS077127\_contig\_number\_7467SRS077502\_contig\_number\_12906SRS077552\_contig\_number\_2288SRS078242\_contig\_number\_30900SRS1041137\_contig\_number\_14681SRS104311\_contig\_number\_28913SRS1055049\_contig\_number\_16254SRS143876\_contig\_number\_27208SRS143895\_contig\_number\_47780SRS144537\_contig\_number\_16982SRS144603\_contig\_number\_16134SRS146812\_contig\_number\_49904SRS147088\_contig\_number\_contig-100\_375.52201


SRS142503\_contig\_number\_46570
2292955
species
1

469590

SRS014923\_contig\_number\_46286SRS063190\_contig\_number\_9640SRS150029\_contig\_number\_3742SRS893383\_contig\_number\_31997
species
4

1

SRS017821\_contig\_number\_30206
2292941
species

2
1739517

SRS022713\_contig\_number\_869SRS076756\_contig\_number\_15339
species

818

SRS017247\_contig\_number\_1231SRS064557\_contig\_number\_10037
species
2

2
species
817

SRS148159\_contig\_number\_23902SRS893342\_contig\_number\_7574

2
species
457390

SRS019397\_contig\_number\_285SRS045244\_contig\_number\_1255

species

SRS022524\_contig\_number\_13169SRS149181\_contig\_number\_3310
338188
2


SRS048164\_contig\_number\_29879SRS062427\_contig\_number\_3635
674529
species
2

6
371601

SRS013800\_contig\_number\_25884SRS019968\_contig\_number\_15071SRS077086\_contig\_number\_3877SRS1055022\_contig\_number\_19282SRS893187\_contig\_number\_17011SRS893378\_contig\_number\_20989
species

species

SRS012273\_contig\_number\_45116SRS012849\_contig\_number\_23970SRS012902\_contig\_number\_517SRS013098\_contig\_number\_38111SRS013215\_contig\_number\_854SRS013382\_contig\_number\_contig-100\_239.22927SRS013476\_contig\_number\_24880SRS014287\_contig\_number\_9919SRS014683\_contig\_number\_12498SRS014855\_contig\_number\_15816SRS014979\_contig\_number\_36170SRS015369\_contig\_number\_3548SRS015578\_contig\_number\_1176SRS015579\_contig\_number\_18539SRS015694\_contig\_number\_16446SRS015794\_contig\_number\_6263SRS015960\_contig\_number\_12515SRS016018\_contig\_number\_1492SRS016056\_contig\_number\_3513SRS016095\_contig\_number\_6360SRS016840\_contig\_number\_1320SRS016897\_contig\_number\_contig-100\_777.26475SRS017191\_contig\_number\_3439SRS017433\_contig\_number\_5670SRS018313\_contig\_number\_9300SRS018427\_contig\_number\_31402SRS018623\_contig\_number\_14602SRS018936\_contig\_number\_12146SRS019068\_contig\_number\_53750SRS019496\_contig\_number\_contig-100\_1295.102386SRS020271\_contig\_number\_contig-100\_477.35524SRS022071\_contig\_number\_34595SRS022137\_contig\_number\_18104SRS023346\_contig\_number\_1596SRS023829\_contig\_number\_319SRS024009\_contig\_number\_1640SRS043001\_contig\_number\_8223SRS043411\_contig\_number\_1862SRS043701\_contig\_number\_11878SRS045004\_contig\_number\_26102SRS045739\_contig\_number\_6889SRS046369\_contig\_number\_15505SRS046717\_contig\_number\_8298SRS049959\_contig\_number\_47991SRS050026\_contig\_number\_contig-100\_1660.74317SRS051031\_contig\_number\_5500SRS053573\_contig\_number\_879SRS054352\_contig\_number\_18634SRS055982\_contig\_number\_18959SRS057478\_contig\_number\_1386SRS058268\_contig\_number\_234SRS062654\_contig\_number\_24940SRS063813\_contig\_number\_contig-100\_48.57279SRS075398\_contig\_number\_866SRS075773\_contig\_number\_19264SRS075821\_contig\_number\_22745SRS077231\_contig\_number\_26588SRS078419\_contig\_number\_19054SRS078665\_contig\_number\_8465SRS097958\_contig\_number\_13921SRS098061\_contig\_number\_14266SRS104084\_contig\_number\_3785SRS1041038\_contig\_number\_14523SRS1041092\_contig\_number\_14198SRS1041095\_contig\_number\_4971SRS1041133\_contig\_number\_contig-100\_280.149004SRS1041134\_contig\_number\_9775SRS104693\_contig\_number\_9881SRS1055034\_contig\_number\_8065SRS1055056\_contig\_number\_contig-100\_154.82994SRS1055095\_contig\_number\_1225SRS140513\_contig\_number\_10926SRS140645\_contig\_number\_386SRS143148\_contig\_number\_16331SRS143372\_contig\_number\_9142SRS143991\_contig\_number\_30282SRS144183\_contig\_number\_42057SRS144297\_contig\_number\_8025SRS147653\_contig\_number\_3489SRS148196\_contig\_number\_contig-100\_583.247782SRS148319\_contig\_number\_16490SRS148511\_contig\_number\_2057SRS148721\_contig\_number\_52334SRS148784\_contig\_number\_17295SRS148970\_contig\_number\_2151SRS149075\_contig\_number\_11607SRS149244\_contig\_number\_2112SRS893231\_contig\_number\_6246SRS893253\_contig\_number\_159SRS893256\_contig\_number\_776SRS893279\_contig\_number\_11326SRS893295\_contig\_number\_10434SRS893300\_contig\_number\_12176SRS893341\_contig\_number\_1688SRS893382\_contig\_number\_1878
28116
95

4
821

SRS015190\_contig\_number\_10654SRS020328\_contig\_number\_27462SRS074964\_contig\_number\_1050SRS1041136\_contig\_number\_10033
species

47678

SRS024132\_contig\_number\_24996SRS053398\_contig\_number\_14369SRS098644\_contig\_number\_10400
species
3

2
820

SRS019693\_contig\_number\_94SRS077024\_contig\_number\_5348
species

species

SRS017103\_contig\_number\_13623
1263054
1

species
2292956

SRS014613\_contig\_number\_2667SRS050801\_contig\_number\_3798SRS076804\_contig\_number\_20874SRS1041039\_contig\_number\_23598SRS142980\_contig\_number\_5477
5

1

SRS104400\_contig\_number\_44170
1841855
species

2

SRS014412\_contig\_number\_17018SRS049773\_contig\_number\_1579
1796613
species

2
46506

SRS023715\_contig\_number\_23563SRS057480\_contig\_number\_contig-100\_81.74924
species
